# Event and Comment

**Published:** 1913-08-15

**Authors:** 


// DENTAL REGISTER.

Vol. LXVII. August 15, 1913. No. 8

### EVENT AND COMMENT.

THE TAGGART PATENT SUIT. Dr. Taggart got a decision in his suit against Dr. B. C. Moll, of Chicago, for infringing his patents on the inlay machine and the process of making the cast inlay. The case was tried in the United States District Court before Judge Landis, and the decision was completely in favor of Taggart on all four of his claims. The defendant, Moll, was restrained from casting inlays or manufacturing a casting machine which the judge ruled was an infringment of the Taggart machine. The defendant was also cited to appear before a master in chancery to make an accounting between the parties interested. This looks like a sweeping victory for Taggart, and if the case is not carried to the higher courts and reversed, it means that Taggart will have the control of the inlay casting process. We have no information at this time as to the future intentions and can therefore make no intelligent comment.

INDEX OF DENTAL LITERATURE. *The Archiv fur Zahnheilkunde* published under the auspices of the American graduates of Europe, has begun the publication of an index of the world's current dental literature as it appears in the nearly two hundred dental magazines published in various parts of the world. It is somewhat of an undertaking, but the well-known author, Dr. Paul de Terra, seems to be the right man for the undertaking. It is not proposed that a digest of all the new articles written shall be made, nor that every so-called original article will be listed, but such as have any cause to be consulted for literary or scientific information will receive such notice as shall call attention of writers and workers to them. It is a prodigious but commendable undertaking, and we trust it will receive generous support by the profession.

DOCTOR OF DENTAL SCIENCE DEGREE. The degree D. D. Sc. has just been established as a higher degree in the Dental Department of the University of Melbourne. This degree was established first in the University of Michigan in 1893, and is conferred only as a post graduate degree and for scientific work in the form of an original research on some dental topic, and is never conferred *in absentia*. At least one full years work is required from the candidate. Since it was established it has been conferred eleven times. The intention is to encourage and reward scientific study, with the hope that it will influence the professional careers of such graduates as may do this work at a time when they can do it to the best advantage, and so prepare themselves with the technique and spirit for scientific research, although they may not take up with teaching or scientific work as a calling. The results have justified the degree at Michigan, other institutions could help on the cause of scientific research in dentistry by a similar action.

FRANK L. PLATT RESIGNS. We regret to learn that Dr. Frank L. Platt, who for many years has so ably edited the *Pacific Dental Gazette*, he has been compelled to discontinue the editorial work that has made his name and the journal he edited a welcome addition to our literary resources. We notice that he is President of the Committee organizing the Pacific Dental Congress, and this probably is the reason for his retirement. We trust he may come back, when that function is successfully concluded, as we confidently expect it to be under his wise and energetic guidance.

